# Evaluation of a digitally-enabled care pathway for acute kidney injury management in hospital emergency admissions

**DOI:** 10.1038/s41746-019-0100-6

**Published:** 2019-07-31

**Authors:** Alistair Connell, Hugh Montgomery, Peter Martin, Claire Nightingale, Omid Sadeghi-Alavijeh, Dominic King, Alan Karthikesalingam, Cian Hughes, Trevor Back, Kareem Ayoub, Mustafa Suleyman, Gareth Jones, Jennifer Cross, Sarah Stanley, Mary Emerson, Charles Merrick, Geraint Rees, Chris Laing, Rosalind Raine

**Affiliations:** 1 Centre for Human Health and Performance, and Institute for Sports, Exercise and Health, University College London, 1st Floor, 170 Tottenham Court Road, London, W1T 7HA UK; 2 DeepMind Health, 5 New Street Square, London, EC4A 3TW UK; 3 Department of Applied Health Research, University College London, 1-19 Torrington Place, London, WC1E 7HB UK; 4 Population Health Research Institute, St George’s, University of London, Cranmer Terrace, London, SW17 0RE UK; 5 Royal Free London NHS Foundation Trust, Pond Street, London, NW3 2QG UK; 6 University College London, Gower Street, London, WC1E 6BT UK; 7 University College Hospital London, Euston Rd, London, NW1 2BU UK

**Keywords:** Acute kidney injury, Outcomes research

## Abstract

We developed a digitally enabled care pathway for acute kidney injury (AKI) management incorporating a mobile detection application, specialist clinical response team and care protocol. Clinical outcome data were collected from adults with AKI on emergency admission before (May 2016 to January 2017) and after (May to September 2017) deployment at the intervention site and another not receiving the intervention. Changes in primary outcome (serum creatinine recovery to ≤120% baseline at hospital discharge) and secondary outcomes (30-day survival, renal replacement therapy, renal or intensive care unit (ICU) admission, worsening AKI stage and length of stay) were measured using interrupted time-series regression. Processes of care data (time to AKI recognition, time to treatment) were extracted from casenotes, and compared over two 9-month periods before and after implementation (January to September 2016 and 2017, respectively) using pre–post analysis. There was no step change in renal recovery or any of the secondary outcomes. Trends for creatinine recovery rates (estimated odds ratio (OR) = 1.04, 95% confidence interval (95% CI): 1.00–1.08, *p* = 0.038) and renal or ICU admission (OR = 0.95, 95% CI: 0.90–1.00, *p* = 0.044) improved significantly at the intervention site. However, difference-in-difference analyses between sites for creatinine recovery (estimated OR = 0.95, 95% CI: 0.90–1.00, *p* = 0.053) and renal or ICU admission (OR = 1.06, 95% CI: 0.98–1.16, *p* = 0.140) were not significant. Among process measures, time to AKI recognition and treatment of nephrotoxicity improved significantly (*p* < 0.001 and 0.047 respectively).

## Introduction

Acute kidney injury (AKI) is a sudden reduction in kidney function, identified and classified by a rise in serum creatinine concentration or reduction in urine output.^1^ Diverse factors contribute to its pathogenesis, including hypovolaemia, sepsis, nephrotoxicity, renal tract obstruction, cardiac or liver dysfunction and primary renal diseases (e.g., glomerulonephritis or interstitial nephritis). AKI affects up to 15% of UK adult hospital admissions,^2,3^ with 50% of cases occurring on presentation in those admitted via the emergency department (ED).^4,5^ AKI causes fluid overload and metabolic derangement, and may adversely affect other organ systems.^6^ It is associated with a need for prolonged hospitalisation,^7^ renal replacement therapy^8^ and high dependency care,^9^ and with greater in-hospital mortality rates.^10^ Lifetime risk of chronic and end-stage kidney disease is raised in AKI survivors,^11^ contributing significantly to their global prevalence.^12^ Excess costs associated with AKI to the National Health Service (NHS) in England exceed £1 billion annually.^3^

AKI management involves recognition, supportive care, therapy directed at the underlying cause, monitoring, renal replacement therapy (if required), appropriate follow-up and interventions to reduce recurrence^6^. Because prompt AKI identification might support timely and effective treatment, NHS England mandated the embedding of an AKI detection algorithm—The NHS Early Detection Algorithm (NHSEDA; Supplementary Fig. 1)—in the laboratory information management systems of English NHS hospitals.^13^ Cases so identified must now be notified to clinicians in results viewing systems.

Direct real-time communication of AKI cases (‘e-alerts’) might be expected to expedite effective treatments. However, in a randomised clinical trial, automated AKI e-alerts delivered via mobile text-messaging yielded no improvement in care processes (rates of renal consultation, contrast and other nephrotoxin administration) or clinical outcome (a composite endpoint of relative maximum change in creatinine, dialysis and death),^14^ perhaps because processes of care were not formally modified. Furthermore, delivering early and specialist care benefits outcomes in other acute conditions,^15,16^ and the UK National Institute for Health and Care Excellence (NICE) recommends that all severe AKI (stage 3) should receive specialist nephrology review, while advocating that the putative benefits of such action be evaluated.^17^

We sought to address these issues through the design and implementation of a novel AKI care pathway in which a specialist response team used a mobile AKI detection and management application and delivered a care protocol. We evaluated the impact of this intervention on patient care and outcomes. Here, we report these for patients presenting acutely to a hospital ED who had AKI on arrival. Results relating to patients developing AKI during the course of their hospital stay, and results from qualitative and economic analyses, will be published separately.

## Results

### Evaluation cohort

The pathway was implemented at the Royal Free Hospital (RFH), a large central London (UK) tertiary referral hospital. The comparator site (which did not receive the digitally enabled care pathway)—Barnet General Hospital (BGH)—is a district general hospital and also part of the Royal Free London NHS Foundation Trust (RFLFT).

At the intervention site (RFH), clinical validation of the 4392 and 2254 AKI alerts during pre-deployment (May 2016 to January 2017) and post-deployment (May to September 2017) phases, respectively, yielded 1760 and 919 AKI episodes in each phase, with 755 (42.9%) and 439 (47.8%) located in the ED. In the pre-deployment and post-deployment phases at BGH, clinical validation of the 2866 and 1364 alerts, respectively, yielded 1669 and 772 AKI episodes, with 1015 (60.8%) and 422 (54.7%) being located in the ED.

Table 1 summarises sociodemographic and clinical characteristics of patients producing AKI alerts in the ED at both evaluation sites and time periods. RFH patients were younger (median 71 vs. 78 years, *p* < 0.001), less deprived (*p* < 0.001) and less likely to be white (65.6 vs. 78.6%, *p* < 0.001) than at BGH. RFH patients had significantly more comorbidity (median and interquartile range (IQR) Charlson score 4.5 (IQR 3.0–7.0) vs. 4.0 (IQR 3.0–6.0), *p* < 0.001) and significantly more severe AKI (*p* < 0.001). The proportion of patients with pre-existing renal disease was also higher (34.1 vs. 19.8%, *p* < 0.001). Comparing the pre- and post-intervention cohorts, there were some significant differences within each evaluation site. In the post-intervention period at RFH, patients were younger (median age 72 vs. 69 years, *p* = 0.003). At BGH, patients in the post-intervention period were less likely to be white (80.8 vs. 73.2%, *p* = 0.030), and had a significantly higher burden of co-morbid disease (*p* < 0.001). At both RFH (37.8 vs. 32.0%, *p* = 0.047) and BGH (23.2 vs. 18.4%, *p* = 0.045), patients in the post-intervention period had a higher burden of pre-existing renal disease.

**Table 1:** Sociodemographic and clinical characteristics of patients producing AKI alerts in the Emergency Department

Variable		Hospital site/time period				*P* value		
		RFH pre	RFH post	BGH pre	BGH post	RFH pre vs. RFH post	BGH pre vs. BGH post	All RFH vs. all BGH
No. of AKI alerts		766	439	1015	422			
Alert severity	AKI1	455 (59.4%)	272 (62.0%)	658 (64.8%)	289 (68.5%)	0.681	0.322	<0.001
	AKI2	161 (21.0%)	86 (19.6%)	210 (20.7%)	83 (19.7%)			
	AKI3	150 (19.6%)	81 (18.5%)	147 (14.5%)	50 (11.8%)			
Male		417 (54.4%)	244 (55.6%)	521 (51.3%)	219 (51.9%)	0.747	0.890	0.092
Median age in years (IQR)		72.00 (59.00–83.50)	69.00 (55.00–82.00)	78.00 (64.00–87.00)	78.00 (67.00–86.00)	0.003	0.793	<0.001
Ethnicity	White	509 (66.4%)	280 (63.8%)	820 (80.8%)	309 (73.2%)	0.739	0.030	<0.001
	Black or Black British	68 (8.9%)	46 (10.5%)	31 (3.1%)	19 (4.5%)			
	Asian or Asian British	79 (10.3%)	53 (12.1%)	75 (7.4%)	46 (10.9%)			
	Mixed	10 (1.3%)	6 (1.4%)	4 (0.4%)	3 (0.7%)			
	Other ethnic groups	100 (13.1%)	54 (12.3%)	85 (8.4%)	45 (10.7%)			
Index of multiple deprivation	Quintile 1 (least deprived)	180 (23.5%)	95 (21.6%)	76 (7.5%)	39 (9.2%)	<0.001	0.898	<0.001
	Quintile 2	191 (24.9%)	100 (22.8%)	212 (20.9%)	88 (20.9%)			
	Quintile 3	183 (23.9%)	96 (21.9%)	315 (31.0%)	122 (28.9%)			
	Quintile 4	169 (22.1%)	112 (25.5%)	305 (30.0%)	112 (26.5%)			
	Quintile 5 (most deprived)	38 (5.0%)	28 (6.4%)	102 (10.0%)	58 (13.7%)			
	Unknown	5 (0.7%)	8 (1.82%)	5 (0.5%)	3 (0.7%)			
Charlson score	0	48 (6.3%)	45 (10.3%)	78 (7.7%)	16 (3.8%)	0.619	<0.001	<0.001
	1	45 (5.9%)	21 (4.78%)	73 (7.2%)	19 (4.5%)			
	2	77 (10.1%)	36 (8.2%)	84 (8.3%)	44 (10.4%)			
	3	93 (12.1%)	43 (9.79%)	137 (13.5%)	57 (13.5%)			
	4	130 (17.0%)	60 (13.7%)	307 (30.2%)	91 (21.6%)			
	≥5	373 (48.7%)	234 (53.3%)	336 (33.1%)	195 (46.2%)			
Pre-existing renal disease present		245 (32.0%)	166 (37.8%)	187 (18.4%)	98 (23.2%)	0.047	0.045	<0.001

*AKI* acute kidney injury, *IQR* interquartile range, *RFH* Royal Free Hospital, *BGH* Barnet General Hospital, *pre* May 2016 to January 2017, *post* May 2017 to September 2017

### Primary outcome

**Fig. 1:**
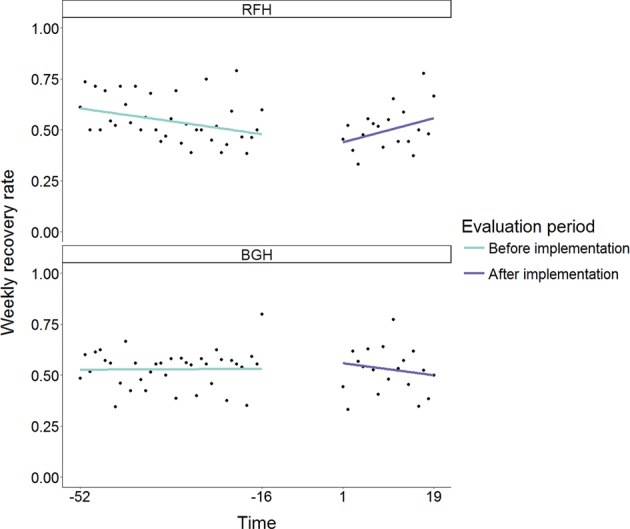
Weekly recovery rate at RFH and BGH before and after implementation of the care pathway. RFH Royal Free Hospital, BGH Barnet General Hospital. Individual data points reflect the rate of each outcome for a single week. Solid lines indicate fitted values from the modelling functions

We found no evidence for a step change in renal recovery rate following the intervention at RFH. The estimated odds ratio (OR) for the intervention step change was 1.03 (95% confidence interval (95% CI): 0.56–1.87), which was not significantly different from 1 (*p* = 0.932). There was also no evidence for a difference in step change of recovery rate between RFH and BGH (estimated OR = 1.10, 95% CI: 0.48–2.53, *p* = 0.830). Estimates from the segmented regression analysis of weekly renal recovery rate which relate to the research hypothesis are shown in Table 2; all model coefficients are shown in Supplementary Table 2. The data and model predictions are illustrated in Fig. 1.

**Table 2:** Results of segmented regression analyses

	Renal recovery				Mortality			
	β	*P* value	OR	95% CI	β	*P* value	OR	95% CI
Intervention	0.03	0.932	1.03	(0.56–1.87)	−0.82	0.055	0.44	(0.19–1.01)
Site × intervention	0.09	0.830	1.10	(0.48–2.53)	−0.66	0.273	0.52	(0.16–1.67)
Time × intervention	0.04	0.038	1.04	(1.00–1.08)	−0.05	0.104	0.95	(0.90–1.01)
Time × site × intervention	−0.05	0.053	0.95	(0.90–1.00)	0.04	0.382	1.04	(0.95–1.13)

	Progression of AKI stage				Admission to ICU/renal unit			
	β	*P* value	OR	95% CI	β	*P* value	OR	95% CI
Intervention	0.19	0.783	1.22	(0.30–4.89)	0.23	0.568	1.26	(0.57–2.79)
Site × intervention	−0.52	0.596	0.59	(0.08–4.08)	0.33	0.597	1.40	(0.40–4.81)
Time × intervention	−0.07	0.162	0.93	(0.83–1.03)	−0.05	0.044	0.95	(0.90–1.00)
Time × site × intervention	0.05	0.467	1.05	(0.92–1.22)	0.06	0.140	1.06	(0.98–1.16)

	Readmission at 30 days				RRT use at 30 days			
	β	*P* value	OR	95% CI	β	*P* value	OR	95% CI
Intervention	0.47	0.204	1.59	(0.78–3.28)	−0.68	0.405	0.51	(0.10–2.50)
Site × intervention	−0.54	0.334	0.58	(0.19–1.73)	−17.24	0.996	0.00	(0.00–Inf)
Time × intervention	0.03	0.195	1.03	(0.99–1.08)	−0.11	0.057	0.90	(0.80–1.00)
Time × site × intervention	0.02	0.552	1.02	(0.95–1.10)	0.07	1.000	1.07	(0.00–476.75)

The coefficient *intervention* provides an estimate of the difference in outcome between the intervention period and the pre-intervention period at RFH. The two-way interaction *site* *×* *intervention* provides an estimate of the difference-in-difference between the two hospital sites. The two-way interaction *time* *×* *intervention* provides an estimate of the difference in outcome trend over time in the intervention period compared to the pre-intervention period at RFT. The three-way interaction *time* *×* *site* *×* *intervention* provides an estimate of the difference-in-difference in the trend between the sites

*OR* odds ratio, *CI* confidence interval, *AKI* acute kidney injury, *ICU* intensive care unit, *RRT* renal replacement therapy

The model estimated a statistically significant change in the trend of renal recovery rates at RFH (estimated OR = 1.04, 95% CI:1.00–1.08, *p* = 0.038), indicating that the trend in the intervention period at RFH was stronger in the direction of higher recovery rates, compared to the pre-intervention period. However, we found no significant difference in the trend change between sites (estimated OR = 0.95, 95% CI: 0.90–1.00, *p* = 0.053). There may have been a trend towards decreasing recovery rates at RFH in the pre-intervention period, which may have been reversed in the intervention period (Fig. 1 and Supplementary Table 2). Model estimates from the sensitivity analysis controlling for differences in casemix do not differ substantially from the primary analysis model estimates (Supplementary Table 3). However, in the sensitivity analysis, none of the four effects of interest are statistically significant.

### Secondary clinical outcomes

Of the 20 coefficients of interest for secondary outcomes, 11 had estimated odds ratios suggesting a beneficial effect of the intervention. The only statistically significant finding was the estimate for the effect of the intervention on the trend change in admission to intensive care unit (ICU) or renal units during RFH admission (estimated OR = 0.95, 95% CI: 0.90–1.00, *p* = 0.044). However, we found no significant difference in the trend change between sites (OR = 1.06, 95% CI: 0.98–1.16, *p* = 0.140). Overall, there was therefore no compelling evidence for an effect of the intervention on these secondary outcomes. Estimates of interest from the segmented regression analyses of secondary outcomes are shown in Table 2. All model coefficients are shown in Supplementary Table 2. The data and model predictions are shown in Supplementary Figs 5 to 9.

At RFH, the median (and IQR) time to renal recovery was 2 days (IQR 1–12 days) before and 3 days (IQR 1–13.25 days) after the introduction of the intervention (*p* = 0.128). At BGH, the median (IQR) time to renal recovery was 2 days (1–9 days) before and 2 days (1–5 days) after the intervention respectively (*p* < 0.001). Using competing risk analyses, a significant reduction in length of stay was demonstrated at both RFH (*p* = 0.024) and BGH (*p* < 0.001) after the RFH implementation period (Supplementary Figs 10 and 11).

### Processes of care

For alerts produced for patients in the ED during the intervention period at RFH, the median (IQR) time from alert generation to alert review by a specialist was 11.50 (IQR 1.00–58.25) min.

**Fig. 2:**
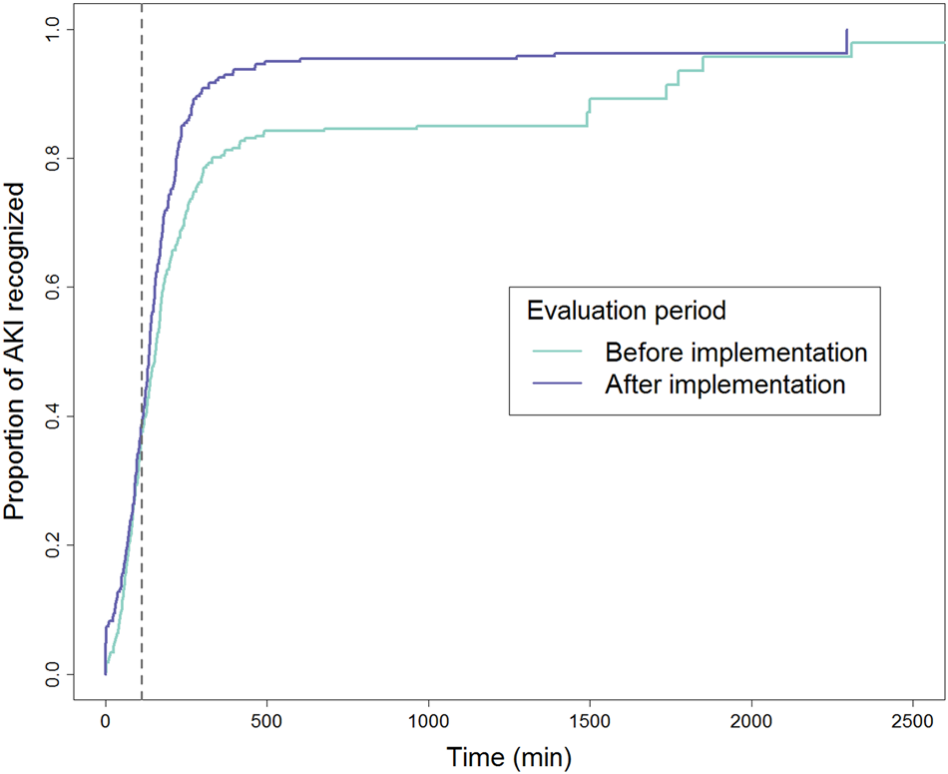
Time to recognition of acute kidney injury (AKI). Kaplan–Meier curves for recognition of AKI after entry to the Emergency Department, before and after the implementation of the care pathway. The vertical dashed line represents the median time of creatinine result release across both time periods

Clinical notes for 540 episodes of clinician-confirmed AKI were reviewed. Of these, 32 were removed from the final analysis due to incomplete data, leaving 266 and 242 in the pre- and post-implementation periods, respectively. Before and after the introduction of the care pathway, the number of unrecognised AKI cases reduced significantly from 33 to 8 (12.4% to 3.3%, *p* < 0.001). Following pathway implementation, time from ED registration to AKI recognition also reduced significantly (log-rank test *p* < 0.001, Fig. 2).

To determine whether improvements in the time to AKI recognition related to changes in the ED admission pathway at RFH, the times of all alerts generated for patients in ED at RFH during the calendar-matched time periods were compared. There were no significant differences in the time at which creatinine tests were released by the laboratory at RFH; the median (IQR) time from entry to ED to AKI alert generation was 113.5 (81.3–155.2) and 107.3 (77.3–141.4) min before and after the introduction of the intervention, respectively (*p* = 0.263). These data suggest improvement in the recognition of AKI related to results viewing in-app, interpretation and timely documentation.

Timeframes for treatment of each of the main causes of AKI, before and after pathway implementation, are detailed in Table 3. At RFH, implementation was associated with a significant reduction in the time to treatment of nephrotoxins (median time to treatment 207.5 vs. 145.0 min, *p* = 0.047). Implementation was associated with faster treatment in patients admitted with sepsis-related AKI and those with obstruction (median times to treatment 114.0 vs. 100.0 min, *p* = 0.288, and 268.0 vs. 224.0 min, *p* = 0.498, respectively), although in both cases the differences were not statistically significant.

**Table 3:** Timeframes for the treatment of AKI at RFH

	Time period	Number of patients treated	Median (IQR) time to treatment (min)	*P* value
Sepsis, infection and hypovolaemia	Before implementation	223	114.0 (50.0–216.5)	0.288
	After implementation	196	100.0 (45.0–195.2)	
Nephrotoxicity	Before implementation	28	207.5 (145.8–313.5)	0.047
	After implementation	43	145.0 (105.5–224.5)	
Obstruction	Before implementation	27	268.0 (186.5–632.5)	0.498
	After implementation	31	224.0 (114.5–875.5)	
Primary renal disease	Before implementation	8	515.5 (203.8–1295.5)	0.345
	After implementation	6	1087.0 (537.0–1602.0)	

*AKI* acute kidney injury, *RFH* Royal Free Hospital*, IQR* interquartile range

## Discussion

We successfully implemented a digitally enabled AKI care pathway and evaluated its impacts using interrupted time-series analysis. There was no evidence of a step change in primary and secondary outcomes at the intervention site following implementation. There was a significant improvement in outcome trend in renal recovery and renal unit/ICU admission at the intervention site, although this trend change was not statistically significantly different from that observed at the comparator site. Pathway implementation was associated with significant improvement in the reliability of AKI recognition, a reduction in the timeframe in which recognition occurred, and a reduction in the timeframe in which adjustment of potentially nephrotoxic medications occurred for this group. Observed improvements in the time to treatment for sepsis and renal tract obstruction were not statistically significant.

Our implementation of the digitally enabled care pathway fulfills NHS England’s objective to achieve earlier diagnosis and more reliable treatment through the use of the NHSEDA. Our evaluation also helps to clarify why e-alerting alone might fail to improve outcomes;^14^ we demonstrate the need to consider the organisational as well as the technical aspects of digital interventions by coupling the alerting system to specific management pathways. However, we were unable to establish definitively whether early specialist input via the digitally enabled pathway improves outcome. There are several possible explanations for these findings.

Firstly, clinical outcome may not be readily modifiable for ED patients at the point of AKI diagnosis. Creatinine-based AKI diagnosis necessarily occurs some time after an insult^18^ and, for ED patient cohorts, both insult and renal injury may also have been established some considerable time before admission. Secondly, baseline processes and outcome at the intervention site were already good: 30-day mortality for pre-intervention patients at RFH was 15.0%, compared to a nationally reported 30-day mortality for all AKI reported to the Renal Registry from April 2016 to September 2017 of 18.1%.^19^ Thirdly, demonstrable process improvement may have been insufficiently impactful on the outcomes considered here. Fourthly, the lack of difference in changes in outcomes between the two sites may have related to parallel improvement initiatives occurring at the comparator site during the study period, including a sepsis improvement project in the ED and an active deteriorating patients improvement programme. It is also possible that the existence of a high-profile improvement initiative in its sister hospital improved AKI awareness in BGH. Finally, we may have lacked statistical power to detect subtle improvements in outcomes, if they existed. These may have been limited to specific patient groups (e.g., patients with severe AKI).

Nonetheless, our data are consistent with recent reports suggesting some benefits of e-alerting systems. A small randomised study in China demonstrated that e-alerting alone increased AKI recognition and specialist nephrology review when applied to intensive care and specialist cardiovascular units.^20^ That this might improve outcomes is supported by a Korean study, which reported the impacts of an e-alert system which facilitated nephrology consultation, comparing data from implementation to historical controls in the same site. AKI detection improved, nephrology consultations within 3 days increased, and the odds of an AKI 2 or 3 event were reduced and of AKI recovery increased. However, the baseline demographic characteristics of the two groups in this study were profoundly different across a multitude of domains.^21^ Two single-site before and after studies of AKI e-alerts were conducted in England. The first—an evaluation of a complex intervention consisting of an AKI specialist team, a ‘Priority Care Checklist' and targeted education activities—was associated with improved AKI detection, fluid assessment and drug assessment, and key general aspects of care. AKI incidence, case length of stay and time to recovery all reduced. However, in common with our research, it is unclear which component(s) influenced the outcomes.^22^ The second study combined an e-alert (telephoned to the ward, but visible to the outreach team) with a protocol for patient management, staff education and a dedicated outreach team. This resulted in reduction in AKI case mortality and length of stay. However, the number of AKI cases detected rose dramatically after the intervention (and continued to do so thereafter), raising the possibility that these impacts were due to the inclusion of patients at lower risk in the denominator group. This would be supported by the fact that coding data (reliant on case-record documentation) rather than laboratory data were used for case ascertainment.^23^

A strength of our evaluation was the use of a comparator site—the first study of its kind to do so. The inclusion of this site highlighted both the difficulty in disentangling the ‘active’ components of a complex intervention and the necessity of comparator data to avoid erroneous conclusions about intervention effectiveness. Additionally, we have clinically reviewed all AKI cases ascertained for analysis using NHSEDA and validated this process.

The limitations of our evaluation include the use of only one intervention site, and the short time periods for data collection. The evaluation design did not allow us to define (or control for) any seasonal changes in outcome; the observed trends could therefore be confounded by seasonal effects. Such effects are known to occur: of 48,457 incident AKI alerts in the Welsh Health Service, 90-day mortality was 28.5% in October–March vs. 25.5% in April–September.^24^ A much longer period of pre-intervention data collection would be required to confirm this; future studies should bear such effects in mind in their design. Additionally, the time-series models we used do not adjust for differences in patient-level variables between sites and time periods. The comparator site hospital control group differed significantly in baseline patient characteristics; these probably related to the complex nature of care at RFH that includes regional cardiac, liver, rheumatology, respiratory, HIV and infectious diseases services as well as a national amyloid service and tertiary vascular, urological cancer and hepatobiliary surgical services; in particular, there was a much higher prevalence of pre-existing renal disease. Our sensitivity analysis controlled for the effects of some potential confounders on renal recovery, and found similar results to our primary analysis, but we cannot rule out that unmeasured confounders may have influenced our findings. Additionally, although time to in-application AKI recognition and virtual review by a specialist was very rapid (median 11.5 min), we were not able to collect comparable data from the pre-implementation phase as this process is new to the care pathway we designed and implemented. The predefined analysis of clinical impact resulted in multiple tests for each outcome we considered, which increased the theoretical risk of rejecting a true null hypothesis. Finally, our initial power calculations did not account for the effect of alert validation on case numbers; weekly case numbers were lower than anticipated at the time we published the protocol.

We have therefore described the successful implementation of a care pathway that enables a team of specialists to be alerted to potential changes in hospitalised patients’ kidney function in real time, rapidly review a curated set of relevant clinical data, intervene proactively and remotely monitor and follow-up cases. We have demonstrated that through such technology, in-application specialist review of AKI cases can take place in minutes. This care pathway has improved the timeliness and reliability of key aspects of AKI care, but definitive conclusions regarding the clinical impact of the pathway cannot be made at this stage and are limited by the scope and nature of our evaluation. Qualitative and economic analyses of the care pathway are currently underway, the results of which will be published in due course, as will data relating to the impact of the care pathway as well as clinical outcomes for patients developing AKI during the course of hospital admission. However, we believe that multi-site evaluation, over longer periods, is required to comprehensively assess the performance and impact on AKI outcomes in different healthcare organisations. Any improvements must also be shown to be of value to users and the wider hospital community, and to be cost effective.

Such digitisation may not only improve delivery of existing AKI care processes, but also enable delivery and evaluation of novel interventions through clinical trials. Advances in machine learning may enable AKI prediction ahead of time, fostering more preventive and targeted therapy. Finally, while the effectiveness of early deployment of specialists might not necessarily be equivalent, key components of this approach may benefit the delivery of time-critical care pathways for other conditions. We believe that any such digitisation must be clinically led and patient-centred, and informed by multi-method evaluation of the organisational, behavioural and technical components of the intervention.

## Methods

### Intervention and comparator sites

The pathway was implemented at the RFH, a large central London (UK) tertiary referral hospital providing a range of acute services—including a 34-bed ICU and a comprehensive inpatient nephrology service. The comparator site (which did not receive the digitally enabled care pathway)—BGH—is a district general hospital providing acute care including a 21-bed intensive care unit (ICU) providing acute renal replacement therapy and liaison nephrology services. Similar arrangements for the early care of AKI patients were in place at both sites prior to pathway implementation. Both are part of the RFLFT.

### The pre-implementation care pathway

Historically, both RFLFT sites had a multidisciplinary educational programme on AKI prevention, recognition and care. AKI was usually managed in its early stages by general acute care and various specialty teams. Using desktop computers, ordering clinicians reviewed test results including serum creatinine, usually in batches at the end of the working day. Results suggestive of AKI were telephoned to the clinical area by laboratory staff and a message placed on the results viewing system, linking to online clinical guidelines. Specialist input into AKI management could be requested through hospital pagers and telephone communication.

### The digitally enabled care pathway

Streams (DeepMind Technologies Ltd, London, UK) is a mobile application deployed on iPhone Operating System (iOS)-enabled smartphones. It processes relevant, routinely collected clinical and demographic data through secure integration with existing information systems. Data security is achieved through on-disk and in-flight encryption, in compliance with NHS Digital information security guidelines. It was first registered with the Medicines and Healthcare Products Regulatory Agency (MRHA) as a Class I, non-measuring, non-sterile medical device on 30 August 2016.

During implementation, Streams analysed serum creatinine results immediately and continuously, alerting the specialist clinical response team to all potential AKI cases as defined by the NHSEDA. Simultaneously, a mobile electronic health record was provided which contained data relevant to AKI management, including a historical trend view of serum creatinine, current AKI stage, specific flags for life-threatening AKI complications, details of previous AKI episodes, demographic information and past medical history (from coded Hospital Episode Statistics data). Videos with detailed demonstrations of app functionality can be found on the DeepMind Health Support website.^25^

A Streams filter excluded paediatric (aged <18 years) patients pre-notification. The RFH specialist clinical response team also excluded patients who would not benefit from the digitally enabled care pathway, i.e., critical care and renal unit inpatients and known dialysis patients.

The specialist clinical response team (henceforth, the ‘AKI response team’) comprised the existing nephrology and ‘patient-at-risk and resuscitation’ (PARRT) teams. The nephrology team comprised a renal consultant and a speciality registrar. Both received all AKI notifications. A renal registrar was onsite 24 h (working in shifts) a day and was usually the first responder. The consultant could triage alerts through secure, remote access if offsite, providing supervision and subsequent clinical review where needed but did not review alerts after midnight unless contacted. The PARRT team are Clinical Nurse Specialists who review at-risk or deteriorating inpatients, 24 h a day, again through shift cover. They received alerts on NHSEDA-defined AKI stages 2 and 3. Through Streams, the AKI response team triaged cases, communicated with other team members and documented clinical reviews and actions undertaken. These digital entries were visible for any subsequent alerts produced for the same patient. Relevant contacts and clinical guidance were available on Streams phones.

Case review within 2 h was suggested for all alerts, although the response team could prioritise patients according to the information available in Streams. Patients with life-threatening complications or deemed at high risk were reviewed immediately. The response team used a care protocol based on existing best practice guidelines^17,26^ (Supplementary Fig. 2). This was annotated and entered into the patient’s notes alongside an advisory sticker for key nursing actions (Supplementary Fig. 3). In general, the response team would support the team primarily responsible for the patient, although the nephrology team would occasionally take over patient care. Streams allowed the response team to monitor AKI recovery remotely in-application. Re-alerting for AKI that had not recovered was enabled 48 h after the first alert. Worsening of AKI stage at any time resulted in a further notification. Secondary response team reviews were undertaken for repeat alerts according to clinical judgement. A diagram outlining the pre- and post-intervention care pathways is provided in Supplementary Fig. 4; while clinical guidelines and specialist response teams existed prior to the new care pathway, implementation aimed to improve the reliability and speed at which AKI recognition and appropriate specialist review occurred.

### Implementation

Prior to deployment, a secure data processing architecture was developed, tested and integrated with existing RFH information systems. The Streams application was developed iteratively, through consultation with the AKI response team. This included formal scoping of user requirements, collaborative workshops with designers and clinicians and user experience testing of the application with mock data. Streams users attended training events and accessed a video users’ guide to both the application and the clinical pathway. During a 16-week pathway optimisation period (January to May 2017), feedback was gathered from the response team and key pathway adjustments made. The optimised care pathway was deployed continuously at RFH from 8 May to 10 September 2017 (18 weeks), during which time the response team comprised 47 users (11 consultants, 21 specialist registrars and 15 PARRT nurses) using six iPhones (Apple Inc., Cupertino, USA).

### Data collection

At both sites, sociodemographic and clinical outcome data from the intervention period (May to September 2017) were compared to data from a pre-deployment phase (May 2016 to January 2017). These data were extracted from the database supporting Streams, and from RFLFT hospital databases. The presence of individual co-morbidities and overall patient-specific Charlson comorbidity index score (which categorises co-morbidities based on the International Classification of Diseases (ICD) diagnosis codes in administrative data) were derived as per Thygesen et al.^27^ Indices of Multiple Deprivation (IMD)—a measure combining seven domains (income/employment/living environment/health/education skills and training deprivation and disability, barriers to housing and services, and crime) into a single deprivation score for a small area—were derived by cross-referencing patient postcodes with the UK Government’s Indices of Deprivation 2015 dataset.^28^ Patients were sorted into quintiles of deprivation (quintile 1 least deprived, quintile 5 most deprived).

All process data were collected as part of an existing RFH project examining processes of care for AKI patients in ED. These data were collected over the two 9-month periods before and after the introduction of the new care pathway (January to September 2016 and 2017, respectively). For each month, 30 clinically validated AKI alerts were selected at random, split evenly across all three stages of AKI severity. Patient records were viewed by a team of RFLFT doctors-in-training unconnected to the project. Times for hospital arrival, AKI recognition (where recognition occurred) and treatment of each principal AKI cause were recorded on a proforma. Recognition was defined as the time at which AKI presence was documented in the patient’s notes. The time at which nephrotoxicity was addressed was defined that at which a physician documented the decision to withhold or adjust the dose of nephrotoxic medication. Times for the treatment of sepsis, hypovolaemia, obstruction and primary renal disease were defined as those recorded in drug chart or procedural documentation. Discrepancies or queries about the cases or data collection methods raised by data collectors were discussed with one author (O.S.-A.), who also reviewed every collected process of care data point. Data collected, and their sources, are detailed in Table 4.

**Table 4:** Definitions of each outcome and sources of data collected

Data category	Measure	Definition	Source of data
Sociodemographic	Age	Age in years at the time of alert	HL7 data aggregated within the Streams data processor
	Gender	Gender codes used in NHS Data Dictionary^36^	HL7 data aggregated within the Streams data processor
	Ethnicity	Ethnicity category codes used in NHS Data Dictionary^36^	HL7 data aggregated within the Streams data processor
	Co-morbid disease	Presence of individual Charlson index co-morbidities and overall Charlson score	HL7 data aggregated within the Streams data processor
	Deprivation	Index of multiple deprivation	Ministry of Housing, Communities & Local Government database
Clinical outcomes	Recovery of renal function	Return to <120% index creatinine (as defined by NHSEDA) by the time of hospital discharge	HL7 data aggregated within the Streams data processor
	Time to recovery of renal function	The time from AKI alert to recovery of renal function (<120% index creatinine).	HL7 data aggregated within the Streams data processor
	Mortality	Death in 30 days following AKI alert	HL7 data aggregated within the Streams data processor
	Progression of AKI stage	Movement between AKI severity classes following AKI alert and prior to hospital discharge	HL7 data aggregated within the Streams data processor
	Admission to high acuity or specialist renal inpatient bed	Admission to acute kidney unit (AKU) or other renal ward, high dependency unit (HDU) or intensive care unit (ICU) during index admission	HL7 data aggregated within the Streams data processor
	Requirement for long-term renal replacement therapy	Use of haemofiltration, haemodiafiltration, haemodialysis or peritoneal dialysis in 30 days following hospital discharge date	RFH Nephrology Clinical Information Management System
	Length of stay	Time from AKI alert to hospital discharge	HL7 data aggregated within the Streams data processor
	Readmission to hospital	Readmission to hospital in 30 days following index admission discharge date	HL7 data aggregated within the Streams data processor
Processes of care	Time to generation of AKI alert	Time (in min) from entry to ED to the alert generation	HL7 data aggregated within the Streams data processor
	Time to AKI alert review	Time (min) from alert generation to alert viewing	HL7 data aggregated within the Streams data processor
	Time to recognition of AKI	Time (min) of documentation of recognition of AKI (in written notes)	Electronic/Paper note review
	Time to treatment	Time of documentation of delivery of antibiotics for sepsis, delivery of fluid for hypovolaemia, relief of obstruction, adjudication of nephrotoxins, and definitive treatment for parenchymal kidney disease	Electronic/Paper note review

Health Level 7 (HL7) messages are used to transfer information between different healthcare IT systems

*AKI* acute kidney injury, *NHS* National Health Service, *NHSEDA* NHS Early Detection Algorithm, *ED* emergency department, *RFH* Royal Free Hospital

### Evaluation of impacts

The study design protocol has been previously published.^29^ The impact of the optimised care pathway on clinical outcomes was assessed by comparing data from the deployment phase (May to September 2017) to data from a pre-deployment phase (May 2016 to January 2017) in both the intervention and comparator sites. The primary outcome was recovery of renal function, defined as a return to a creatinine level within 120% of the baseline (itself defined by NHSEDA) prior to hospital discharge. Predefined endpoints reflecting secondary processes of care and clinical outcomes are outlined in Table 4.

**Fig. 3:**
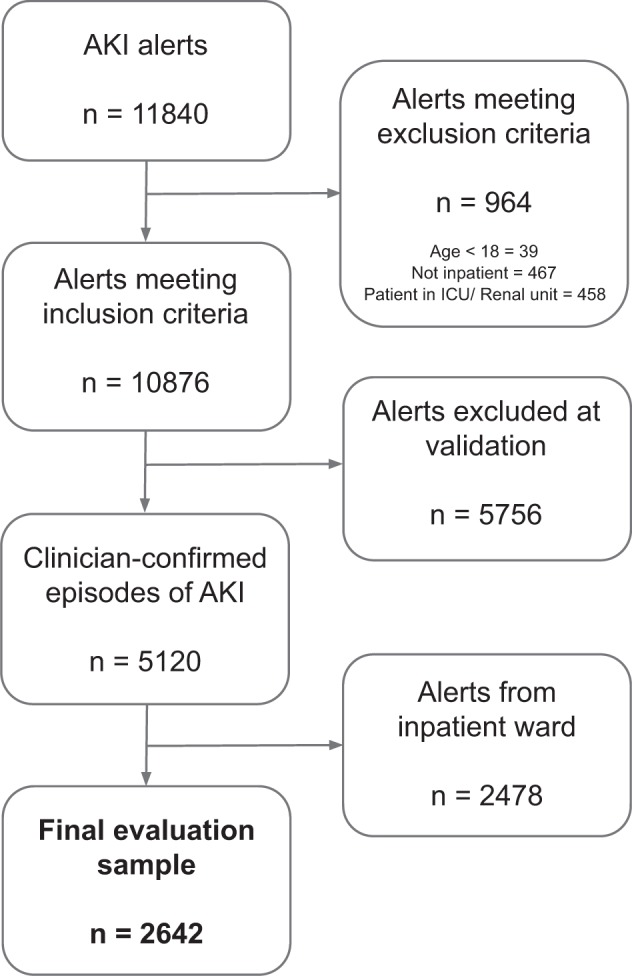
Defining the final evaluation sample

At both sites, NHSEDA was used to identify potential AKI cases. Because the NHSEDA can produce false positives,^30^ two authors (A.C. and C.L.) clinically validated all AKI alerts produced from all time periods at both hospital sites. Only clinician-confirmed episodes of AKI were included in the analysis. In this paper we report the outcomes of patients presenting acutely to RFH and BGH hospital ED who had AKI on arrival during the pre-deployment and deployment phases (Fig. 3).

### Statistical analysis

All data were pseudonymised prior to transfer from the RFLFT to University College London (UCL) for analysis. All analyses were performed using R, version 3.4.3.^31^ Segmented regression analysis was used to estimate the effect of the intervention on our primary outcome—recovery of renal function prior to hospital discharge—and on five secondary outcome measures: mortality within 30 days of alert; progression of AKI stage; transfer to renal/intensive care units during admission; readmission within 30 days of discharge; and dependence on renal replacement therapy 30 days after discharge. All outcomes were measured as weekly proportions. Binomial regression models with a logit link were used. The variable ‘intervention' was coded 1 for the time period after the intervention (May to September 2017) and 0 for the pre-intervention time period (May 2016 to January 2017). The intervention and comparator sites were coded 1 and 0 respectively. The variable ‘time' denoted the week number, with 1 denoting the first week of the intervention period, and weeks in the pre-intervention period being denoted by negative numbers. The statistical model used was:

$$\begin{array}{l}\mathrm {logit}(y) = \beta _0 + \beta _1\mathrm {int} + \beta _2\mathrm {time} + \beta _3\mathrm {site} + \beta _4\mathrm {int} \times \mathrm {time} \\ \qquad \qquad \qquad \ + \beta _5\mathrm {int} \times \mathrm {site} + \beta _6\mathrm {time} \times \mathrm {site} + \beta _7\mathrm {int} \times \mathrm {time} \times \mathrm {site}\end{array}$$

where *y* denotes the proportion of interest, int, time and site denote the variables intervention, time and site, respectively (as defined above), and *β*_0_, …, *β*_7_ are the coefficients to be estimated. We focus on four effects of interest. Two coefficients evaluate the evidence for a step change in the outcome due to the intervention: the effect of *intervention* estimates the step change in outcome at the start of the intervention period at RFH. The interaction *site* *×* *intervention* estimates the difference-in-difference in the step change between the two hospital sites. Two further effects of interest evaluate the evidence for a change in temporal trend in the outcome due to the intervention: the interaction *time* *×* *intervention* estimates of the difference in outcome trend over time between the intervention period and the pre-intervention period at RFL; the three-way interaction *time* *×* *site* *×* *intervention* estimates the difference-in-difference in the trend between the sites. All models were checked for autocorrelation by inspecting the autocorrelation function up to lag 15; no significant autocorrelation was found. At the point of protocol publication, it was not anticipated that we would be able to collect patient-level data relating to sociodemographics and co-morbid disease. To examine the robustness of our primary outcome analysis, we performed a sensitivity analysis using binary logistic regression that used the same model as above, except that the outcome was defined at the patient level and that patient-level characteristics were included as covariates. Covariates used for this model were age, sex, ethnicity category, index of multiple deprivation, AKI alert level, the presence of complications at the time of alert, and the presence of individual Charlson Score co-morbidities. The addition of these covariates allowed us to adjust for any differences in casemix between sites, and within sites over time.

The time to creatinine recovery (where this occurred by hospital discharge) was analysed using the Wilcoxon rank-sum test. A competing risk analysis was performed to estimate the effect of the intervention on the length of hospital stay to allow for the effects of in-hospital death on this outcome.^32^ A survival analysis was performed to determine the effect of the intervention on the time to recognition of AKI. All other processes of care and sociodemographic variables were analysed using the Wilcoxon rank-sum and chi-squared tests as appropriate.

In order to assess the reliability of case validation, 500 alerts selected randomly from all time periods and all sites were validated a second time. Cohen’s kappa coefficient was used to determine intra- and inter-rater reliability (Supplementary Table 1).

### Ethical approval

The digitally enabled care pathway constituted a new standard service at RFH. The UCL Joint Research Office reviewed the study protocol and judged that the project fell under the remit of service evaluation, as per guidance from the NHS Health Research Authority.^33^ As such, no patient consent was required. The evaluation was registered with the RFH Audit Lead and Medical Director. An independent Data Monitoring Committee (which included a patient member) reviewed all analyses prior to preparation for publication. A full list of Committee members is provided as Supplementary Material.

DeepMind was acquired by Google in 2014 and is now part of the Alphabet group. The deployment of Streams at RFH was the subject of an investigation by the Information Commissioner’s Office in 2017. RFH has since published an audit completed to comply with undertakings following this investigation.^34^ In November 2018, it was announced that the Streams team will be joining Google as part of a wider health effort.^35^

### Reporting Summary

Further information on experimental design is available in the Nature Research Reporting Summary linked to this paper.

## Supplementary information

Reporting summary

Supplementary materials

## Data Availability

The datasets generated and analysed during the service evaluation are pseudonymised and will not be made publicly available at the request of RFLFT.

## Supplementary information

**Supplementary information** accompanies the paper on the *npj Digital Medicine* website (10.1038/s41746-019-0100-6).
