# Supplementary material for: Evaluation of a digitally-enabled care pathway for acute kidney injury management in hospital emergency admissions
Source: NPJ Digit Med. 2019 Jul 31;2:67. doi: 10.1038/s41746-019-0100-6 (PMC6669220; doi:10.1038/s41746-019-0100-6)
Supplement: Supplementary file 1 — Reporting summary [file 41746_2019_100_MOESM1_ESM.pdf]

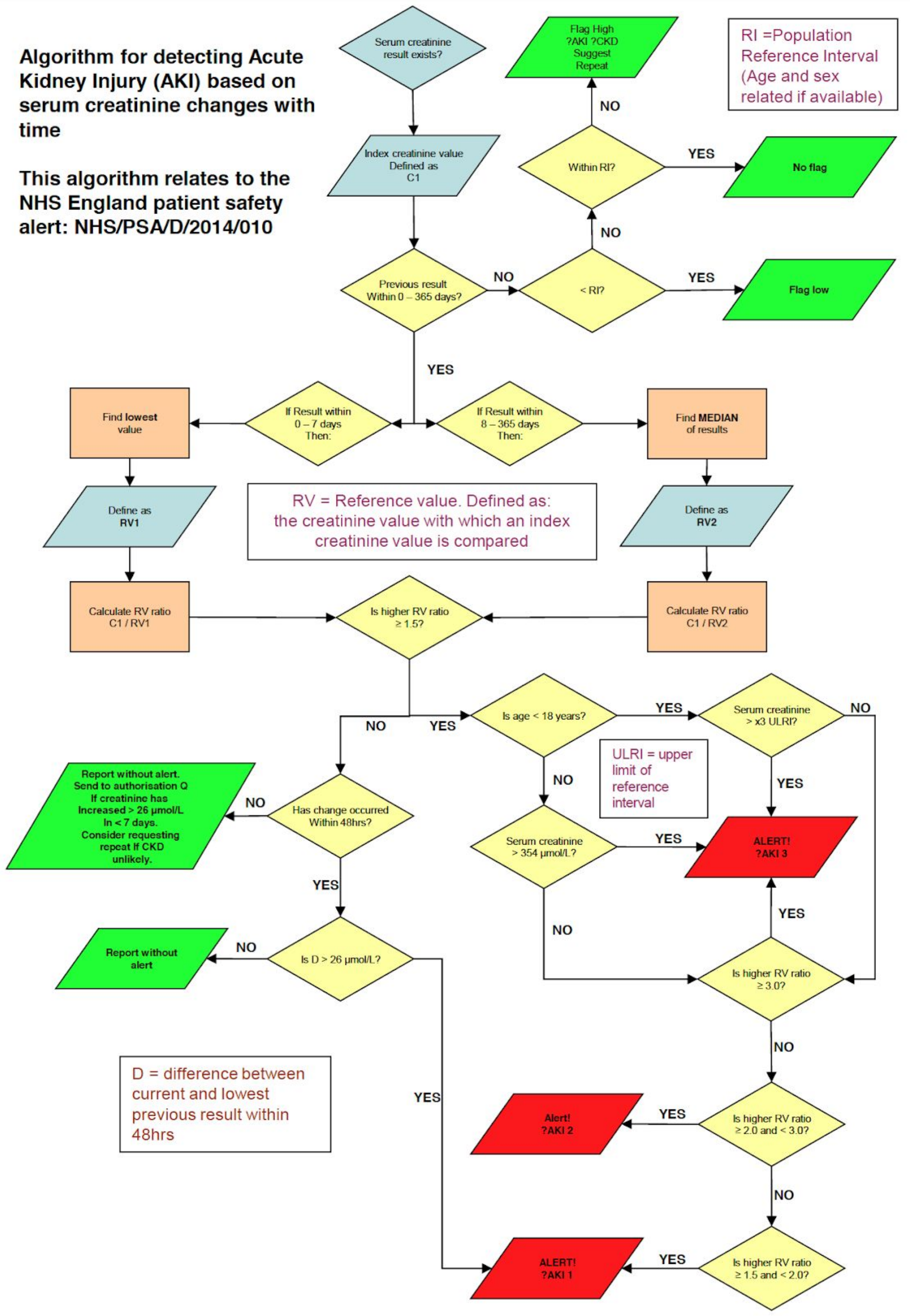

Supplementary figure 1: the NHS Early Detection Algorithm (NHSEDA)

Royal Free London NHS

NHS Foundation Trust

This patient has generated an AKI alert on the Streams application.
The AKI response team are here to support and advise.
Ongoing management remains the responsibility of the home team unless otherwise agreed.

affix patient sticker here

We recommend the following actions to STOP-AKI:

**Management of life threatening complications of AKI**	**Critically ill:** Call PARRT (2525) or ITU (1030)	☐
	**Hyperkalaemia or acidosis:** commence medical therapy as per guidelines	☐
	**Fluid overload:** Commence diuretics, nitrates/oxygen (if necessary), fluid restriction	☐
**Sepsis and hypoperfusion**	**Sepsis:** complete Sepsis 6 care bundle	☐
	**Has an infection causing AKI:** send cultures, commence or escalate antibiotics	☐
	**Hypovolaemic:** Start bolus fluid protocol. Give 500mls crystalloid and reassess, repeat as necessary. Escalate to senior review after 2 litres bolus therapy	☐
	Commence maintenance IV fluids	☐
**Toxicity**	**Drug cessation or adjustment required**	☐
**Obstruction**	**Obstruction is possible and patient needs same day diagnostic renal USS** Please call Matteo Rossi for bedside USS on 07443101848. If out of hours then discuss with radiology (1462). If obstruction present please contact urology registrar on 1487 or on x39536	☐
**Primary Renal Disease**	**Perform urine dipstick**	☐
	If urine dip clear: order 'AKI diagnostic set (basic)' on Cerner	☐
	If blood or protein present: order 'AKI diagnostic set (glomerular)' on Cerner	☐
**General advice**<br><br>If in doubt, contact the AKI registrar on 07950860822 (day) or 07950843257 (night)<br><br>For guidelines and education, visit londonaki.net or download the London AKI app:	- Take 4 hourly observations & ensure an escalation plan is in place<br>- Commence a fluid balance chart, measure weights daily and set a daily fluid balance target<br>- Daily bloods: use 'AKI follow up' order set on Cerner and follow up to renal recovery<br>- Avoid contrast if possible. Consider prophylaxis where contrast absolutely necessary<br>- If renal function does not return to baseline at discharge, contact AKI registrar for advice	
**Follow up**	We will only see if contacted by you or re-alerted in Streams due to worsening AKI	☐
	We will schedule a further review	☐
	We will take over care of patient	☐

TIME SEEN:______:______ DATE: ______/_____/_____ SIGNED:__________________________________

Grade: Registrar ☐ Consultant ☐ NAME:____________________________________

Supplementary figure 2: the care protocol

This patient has developed acute kidney injury (AKI)

You need to do the following to help your patient recover:

Do a urine dipstick

Start 4 hourly observations

Start a fluid balance chart
Ensure prescribed oral or intravenous fluid is administered.
Document inputs/ outputs/ 24 hour fluid balance and weight.

Escalate concerns
If concerned, get help. Call the medical team, and PARRT (2525).

Supplementary figure 3: nursing advisory sticker

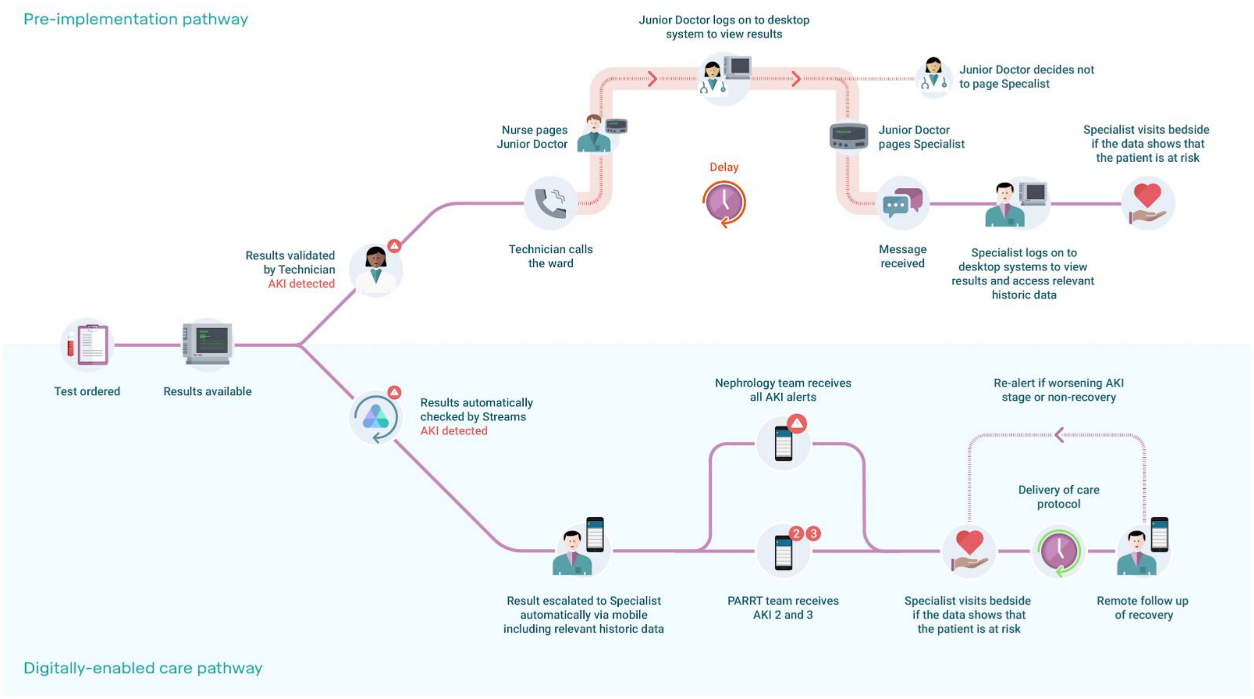

Supplementary figure 4: AKI care pathways before and after the introduction of the digitally-enabled care pathway

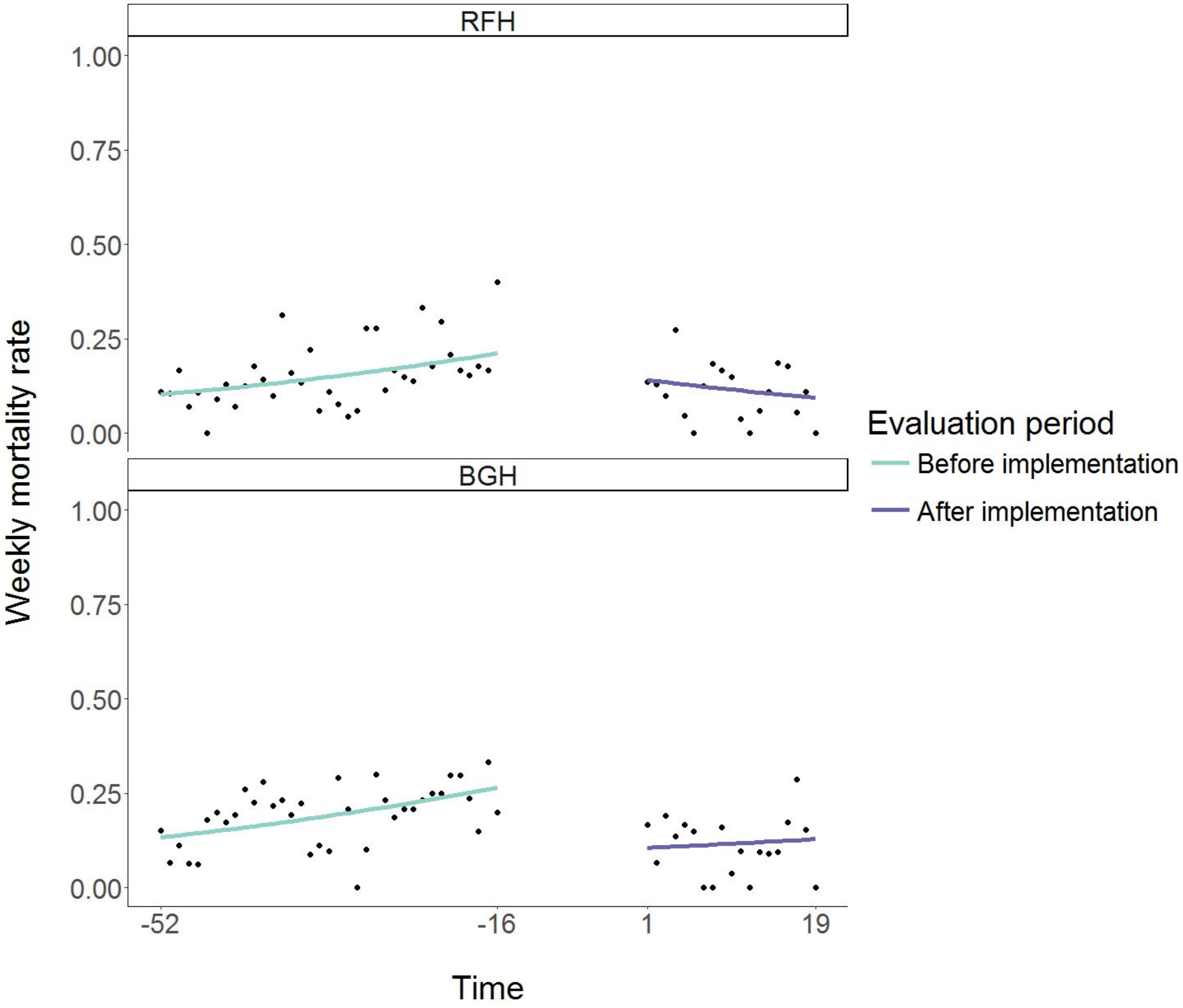

Supplementary Figure 5: Weekly rates of mortality at RFH and BGH before and after implementation of the care pathway

RFH = Royal Free Hospital, BGH = Barnet General Hospital.

Individual data points reflect the rate of each outcome for a single week.

Solid lines indicate fitted values from the modelling functions.

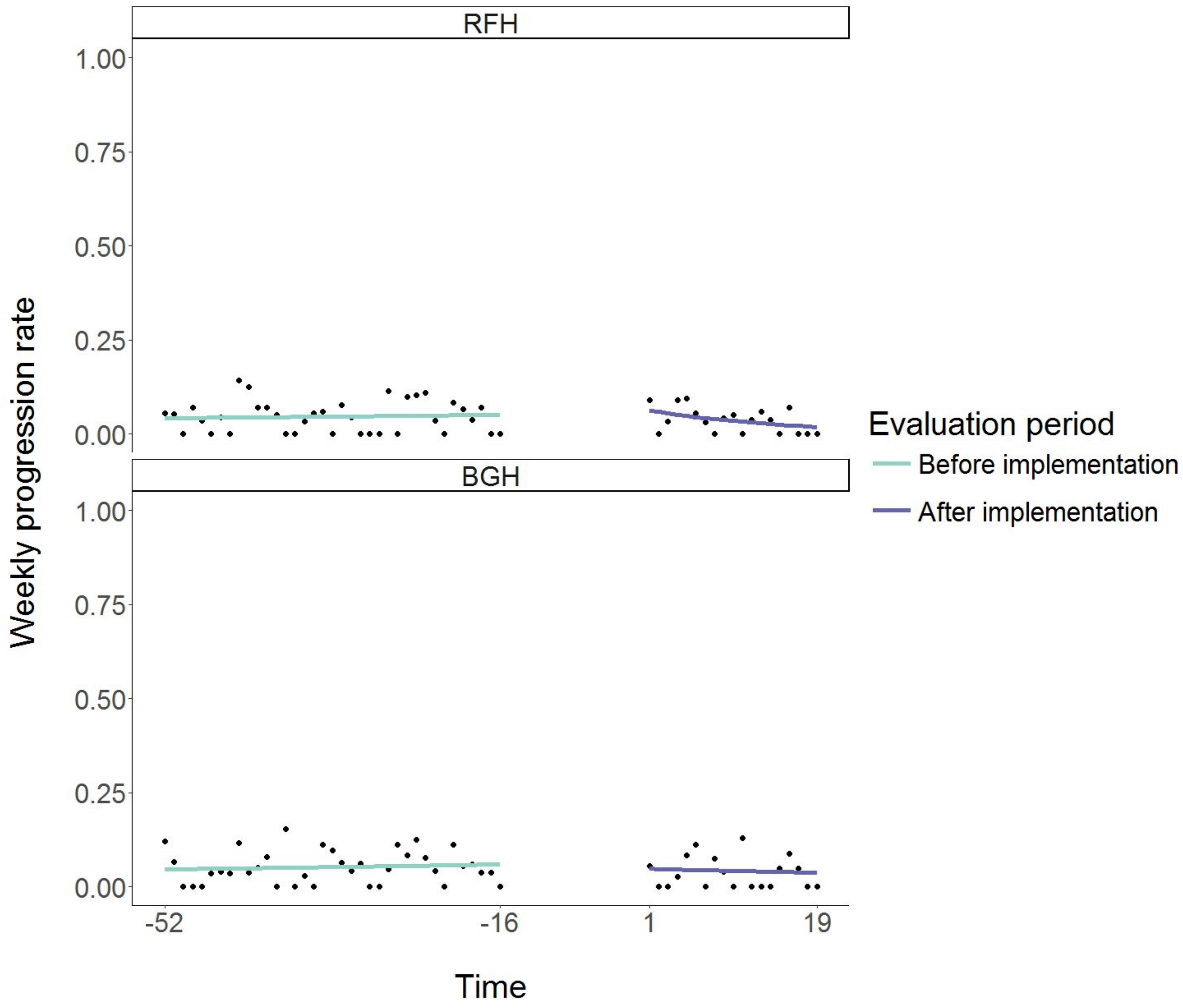

Supplementary Figure 6: Weekly rates of AKI progression at RFH and BGH before and after implementation of the care pathway

RFH = Royal Free Hospital, BGH = Barnet General Hospital.

Individual data points reflect the rate of each outcome for a single week.

Solid lines indicate fitted values from the modelling functions.

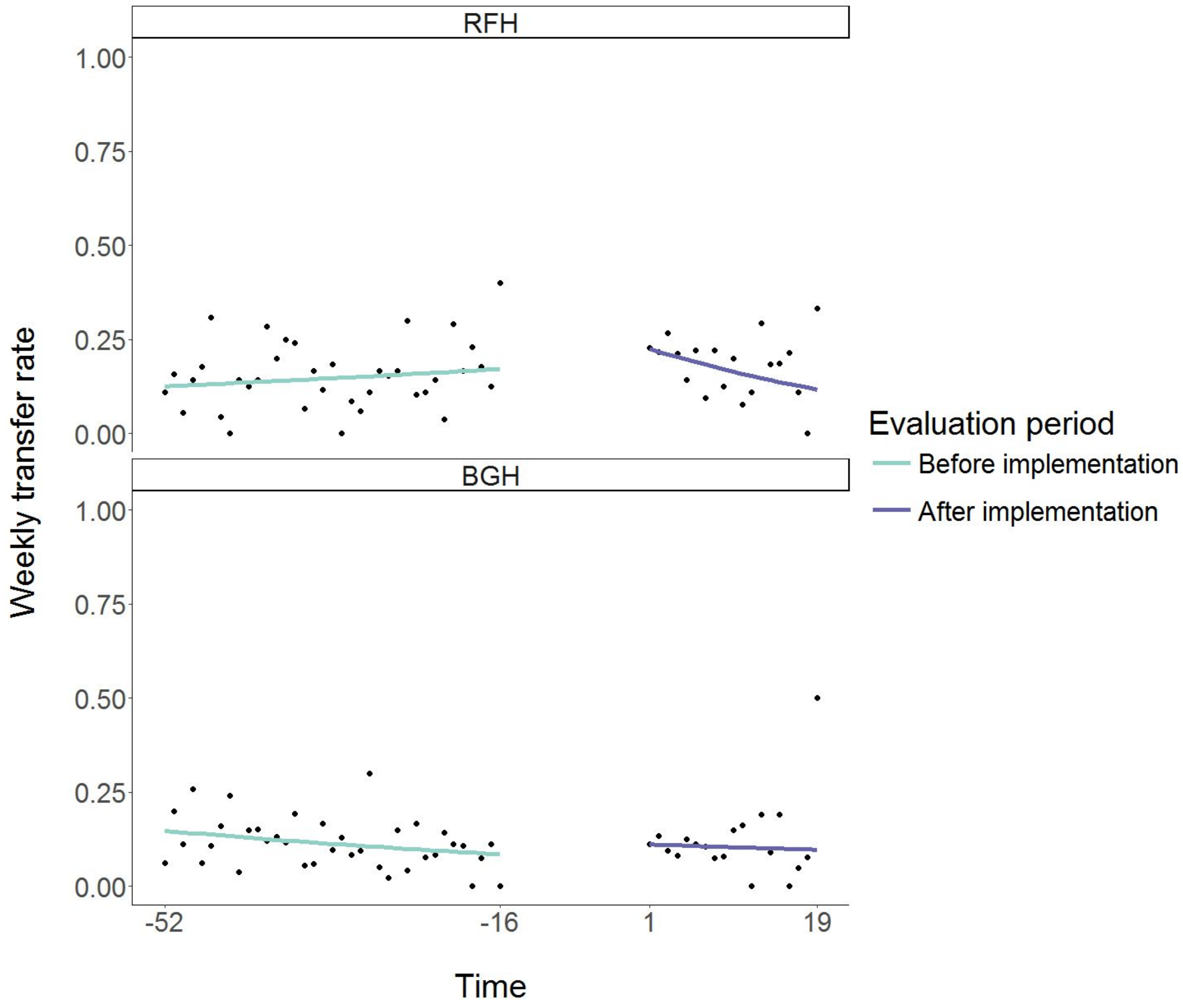

Supplementary Figure 7: Weekly rates of transfer to ITU/ renal unit at RFH and BGH before and after implementation of the care pathway

RFH = Royal Free Hospital, BGH = Barnet General Hospital.

Individual data points reflect the rate of each outcome for a single week.

Solid lines indicate fitted values from the modelling functions.

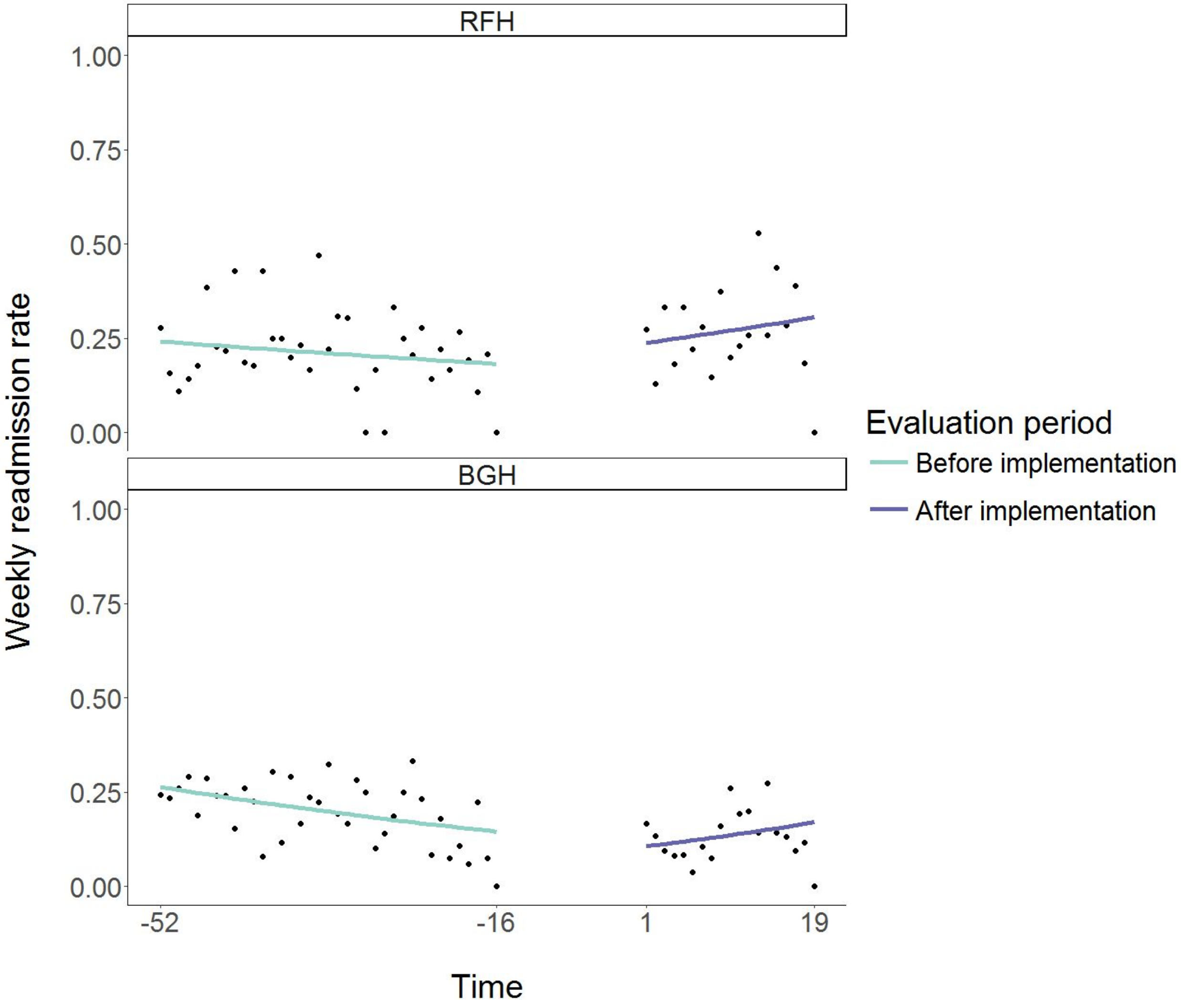

Supplementary Figure 8: Weekly rates of readmission at RFH and BGH before and after implementation of the care pathway

RFH = Royal Free Hospital, BGH = Barnet General Hospital.

Individual data points reflect the rate of each outcome for a single week.

Solid lines indicate fitted values from the modelling functions.

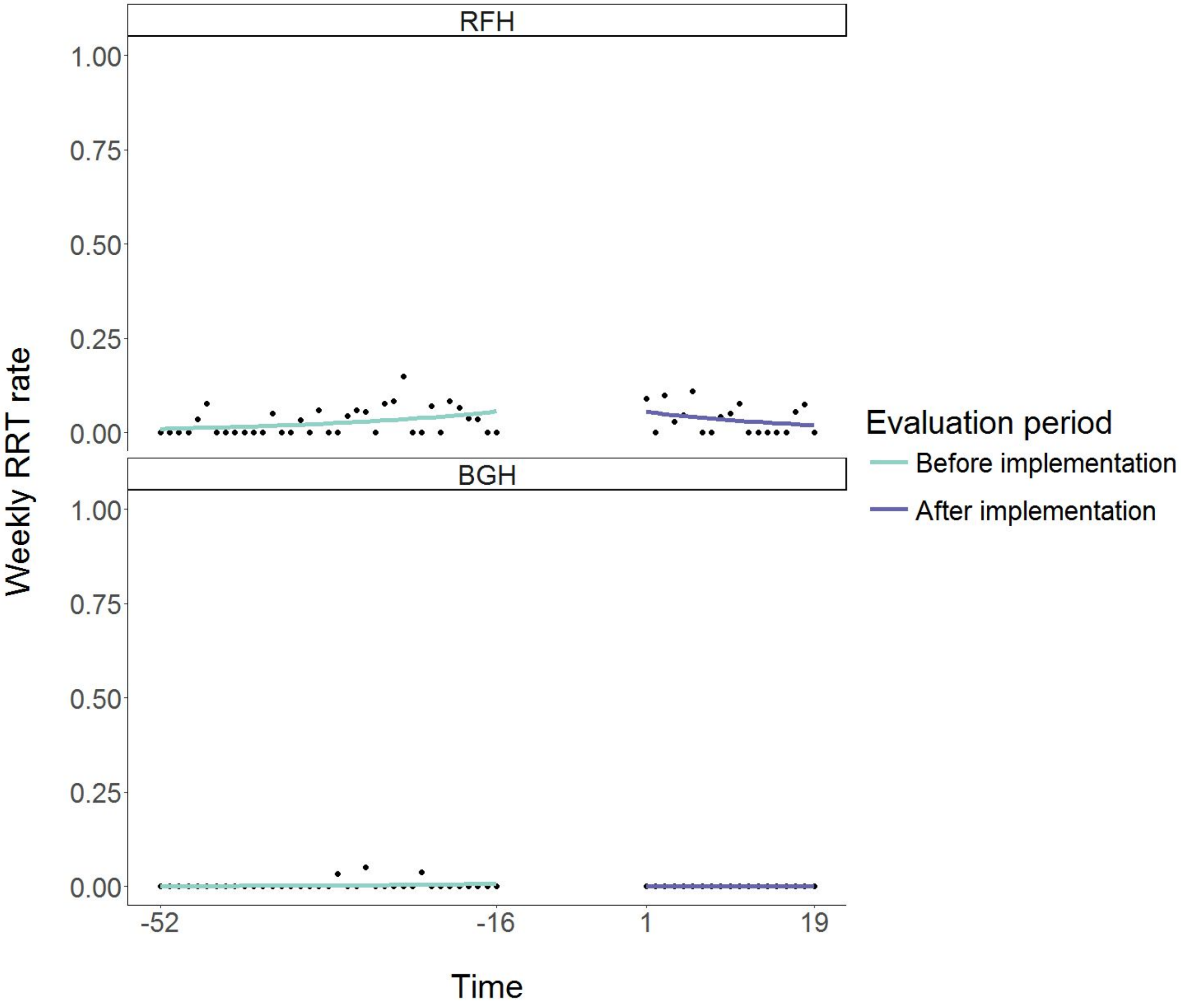

Supplementary Figure 9: Weekly rates of 30-day dependence on renal replacement therapy at RFH and BGH before and after implementation of the care pathway

RFH = Royal Free Hospital, BGH = Barnet General Hospital.

Individual data points reflect the rate of each outcome for a single week.

Solid lines indicate fitted values from the modelling functions.

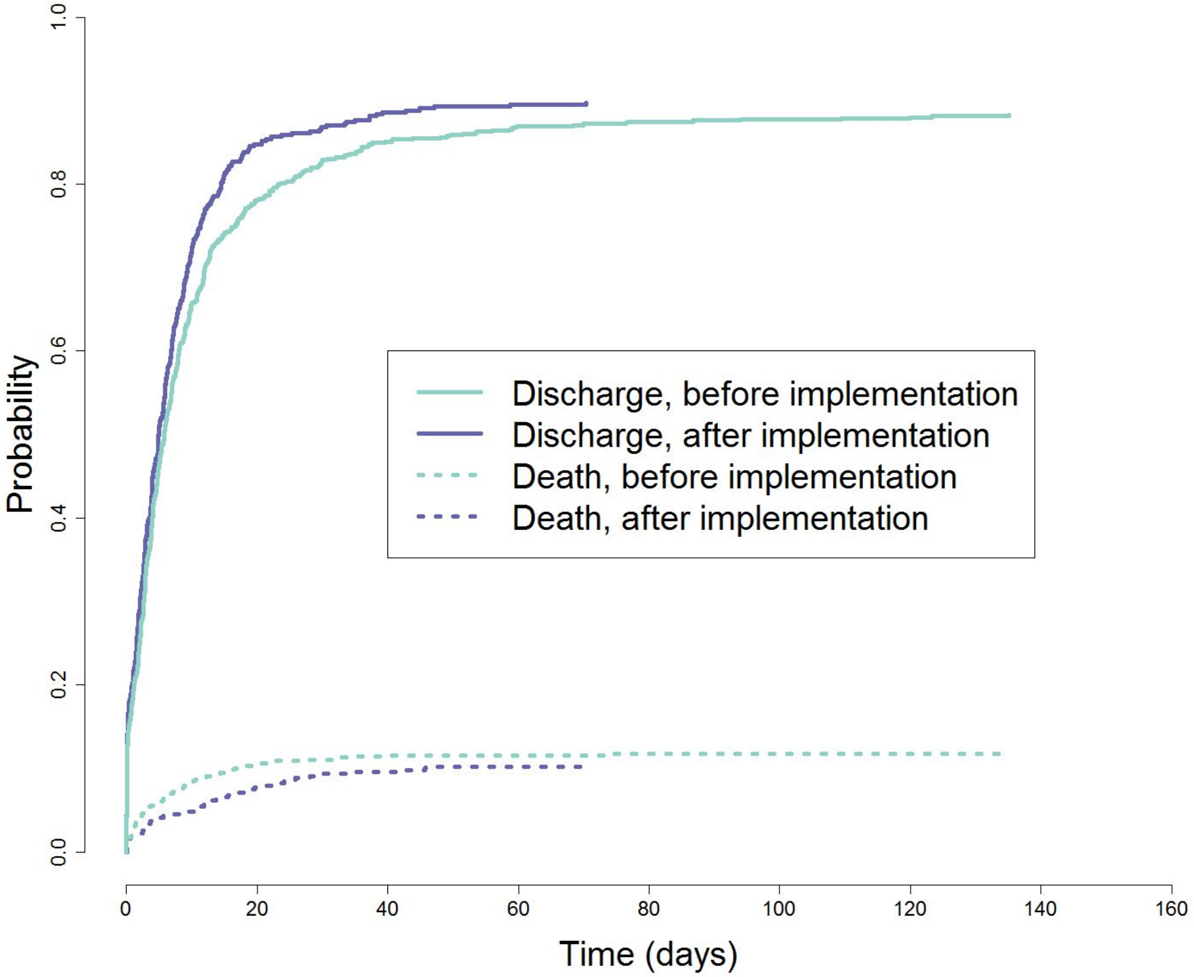

Supplementary figure 10: plot of competing risk analysis for mortality and hospital discharge at RFH

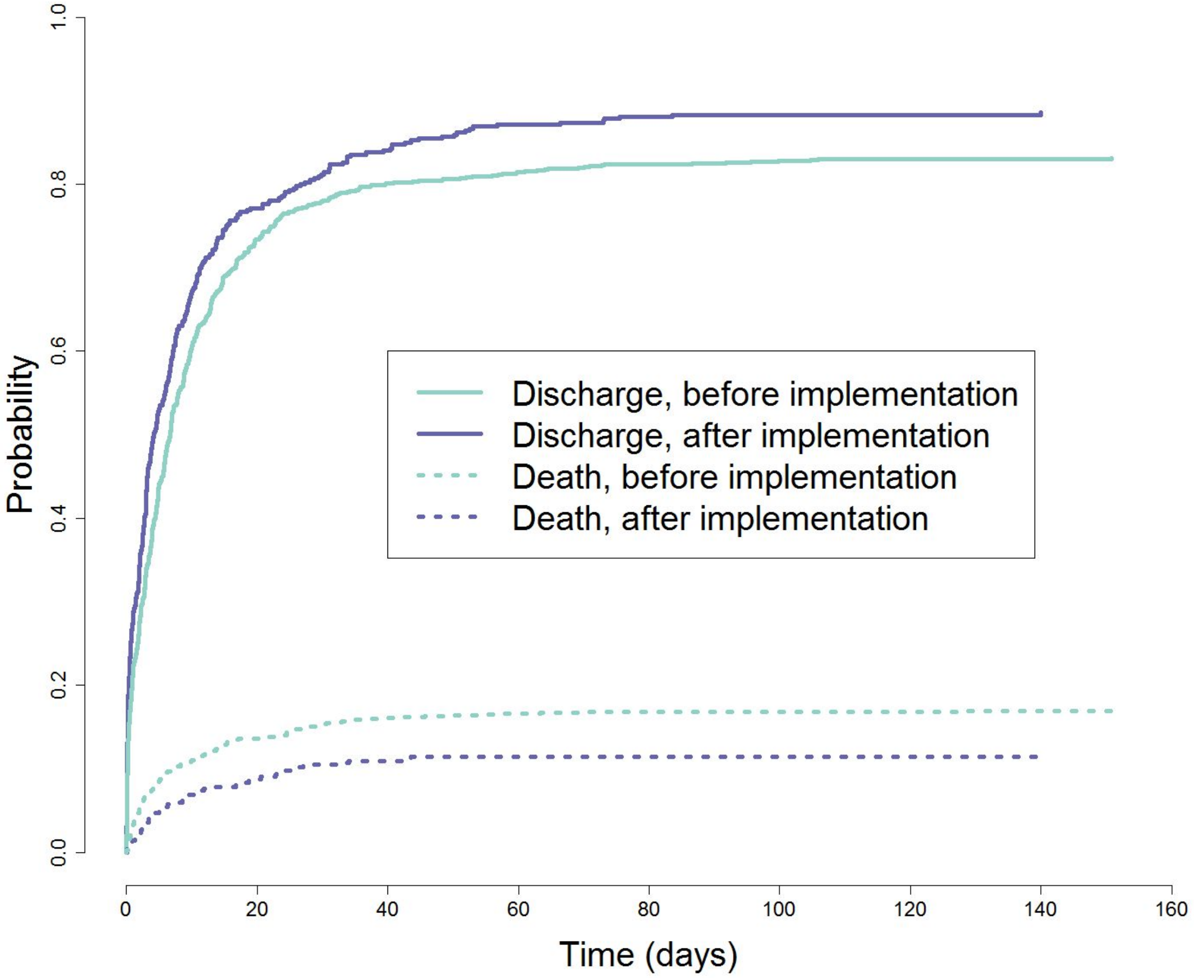

Supplementary figure 11: plot of competing risk analysis for mortality and hospital discharge at BGH

	Operator 1	Operator 2
Operator 1	κ = 0.83 (0.76 - 0.90)	
Operator 2	κ = 0.75 (0.65 - 0.84)	κ = 0.79 (0.71 - 0.87)

Supplementary table 1: Inter- and intra-operator variability analyses

From the pool of alerts, a random selection of 250 from each operator were validated again by both. For each comparison pair, Cohen's kappa coefficient was calculated to establish inter- and intra-operator variability. 95% confidence intervals are shown in brackets

	Renal recovery				Mortality			
	β	p value	OR	95% CI	β	p value	OR	95% CI
intervention	0.03	0.932	1.03	(0.56-1.87)	-0.82	0.055	0.44	(0.19-1.01)
time	-0.01	0.038	0.99	(0.97-1.00)	0.02	0.014	1.02	(1.00-1.04)
site	0.43	0.174	1.54	(0.83-2.85)	0.29	0.481	1.33	(0.60-2.97)
site×intervention	0.09	0.830	1.10	(0.48-2.53)	-0.66	0.273	0.52	(0.16-1.67)
time×intervention	0.04	0.038	1.04	(1.00-1.08)	-0.05	0.104	0.95	(0.90-1.01)
time×site	0.01	0.107	1.01	(1.00-1.03)	0.00	0.987	1.00	(0.98-1.02)
time×site×intervention	-0.05	0.053	0.95	(0.90-1.00)	0.04	0.382	1.04	(0.95-1.13)

	Progression of AKI stage				Admission to ITU/Renal Unit			
	β	p value	OR	95% CI	β	p value	OR	95% CI
intervention	0.19	0.783	1.22	(0.30-4.89)	0.23	0.568	1.26	(0.57-2.79)
time	0.01	0.728	1.01	(0.97-1.04)	0.01	0.295	1.01	(0.99-1.03)
site	0.17	0.807	1.19	(0.29-4.91)	-1.22	0.010	0.30	(0.12-0.75)
site×intervention	-0.52	0.596	0.59	(0.08-4.08)	0.33	0.597	1.40	(0.40-4.81)
time×intervention	-0.07	0.162	0.93	(0.83-1.03)	-0.05	0.044	0.95	(0.90-1.00)
time×site	0.00	0.937	1.00	(0.96-1.04)	-0.03	0.043	0.97	(0.95-1.00)
time×site×intervention	0.05	0.467	1.05	(0.92-1.22)	0.06	0.140	1.06	(0.98-1.16)

	Readmission at 30d				RRT use at 30d			
	β	p value	OR	95% CI	β	p value	OR	95% CI
intervention	0.47	0.204	1.59	(0.78-3.28)	-0.68	0.405	0.51	(0.10-2.50)
time	-0.01	0.236	0.99	(0.97-1.01)	0.05	0.034	1.05	(1.01-1.10)
site	-0.43	0.281	0.65	(0.30-1.43)	-1.98	0.297	0.14	(0.00-5.49)
site×intervention	-0.54	0.334	0.58	(0.19-1.73)	-17.24	0.996	0.00	(0.00-Inf)
time×intervention	0.03	0.195	1.03	(0.99-1.08)	-0.11	0.057	0.90	(0.80-1.00)
time×site	-0.01	0.343	0.99	(0.97-1.01)	0.01	0.892	1.01	(0.89-1.17)
time×site×intervention	0.02	0.552	1.02	(0.95-1.10)	0.07	1.000	1.07	(0.00-476.75)

Supplementary table 2: Results of segmented regression analyses, including all estimated coefficients

The coefficient *intervention* provides an estimate of the difference in outcome between the intervention period and the pre-intervention period at RFH. The two-way interaction *site×intervention* provides an estimate of the difference-in-difference between the two hospital sites. The two-way interaction *time×intervention* provides an estimate of the difference in outcome trend over time in the intervention period compared to the pre-intervention period at RFT. The three-way interaction *time×site×intervention* provides an estimate of the difference-in-difference in the trend between the sites.

	Renal recovery			
	**β**	**p value**	**OR**	**95% CI**
intervention	0.10	0.746	1.11	0.60 - 2.03
site×intervention	-0.03	0.939	0.97	0.42 - 2.25
time×intervention	0.03	0.143	1.03	0.99 - 1.07
time×site×intervention	-0.04	0.140	0.96	0.90 - 1.01

Supplementary table 3: Results from binary logistic regression (sensitivity analysis)

Renal recovery was defined at the patient level and patient-level characteristics were included as covariates. Covariates used for this model were age, sex, ethnicity category, index of multiple deprivation, AKI alert level, the presence of complications at the time of alert, and the presence of individual Charlson Score co-morbidities. The coefficient *intervention* provides an estimate of the difference in outcome between the intervention period and the pre-intervention period at RFH. The two-way interaction *site×intervention* provides an estimate of the difference-in-difference between the two hospital sites. The two-way interaction *time×intervention* provides an estimate of the difference in outcome trend over time in the intervention period compared to the pre-intervention period at RFT. The three-way interaction *time×site×intervention* provides an estimate of the difference-in-difference in the trend between the sites.

RFL Data Monitoring Committee

Chair

Prof. Kevin Moore
Professor of Hepatology, Royal Free Hospital

Patient representative

Michael Wise
Acute Kidney Injury National Programme Board

External members

Sir Nick Black
Professor of Health Services Research, London School of Hygiene and Tropical Medicine

Dr. Neil Ashman
Consultant Nephrologist and Deputy Managing Director, Barts Health

Royal Free Hospital members

Dr. Jim Buckley
Consultant in Intensive Care Medicine

Dr. Nick Murch
Consultant in Acute Medicine

Dr. Jonathan Costello
Clinical Director, Emergency Medicine

Dr. Bimbi Fernando
Consultant in Transplant Surgery

Dr. Penny Smith
Consultant in Acute Medicine and Chief Medical Informatics Officer

Dr. Banwari Agarwal
Consultant in Intensive Care Medicine

Dr. Rupert Negus
Consultant in Acute Medicine and Gastroenterology
